# Discovery of DNA methylation markers in cervical cancer using relaxation ranking

**DOI:** 10.1186/1755-8794-1-57

**Published:** 2008-11-24

**Authors:** Maté Ongenaert, G Bea A Wisman, Haukeline H Volders, Alice J Koning, Ate GJ van der Zee, Wim van Criekinge, Ed Schuuring

**Affiliations:** 1 Laboratory for Bioinformatics and Computational Genomics (BioBix), Department of Molecular Biotechnology, Faculty of Bioscience Engineering, Ghent University, Belgium; 2 Department of Gynaecologic Oncology, University Medical Centre Groningen, University of Groningen, The Netherlands; 3 Oncomethylome Sciences SA, Sart-Tilman (Liege), Belgium; 4 Department of Pathology, University Medical Centre Groningen, University of Groningen, The Netherlands

## Abstract

**Background:**

To discover cancer specific DNA methylation markers, large-scale screening methods are widely used. The pharmacological unmasking expression microarray approach is an elegant method to enrich for genes that are silenced and re-expressed during functional reversal of DNA methylation upon treatment with demethylation agents. However, such experiments are performed in *in vitro *(cancer) cell lines, mostly with poor relevance when extrapolating to primary cancers. To overcome this problem, we incorporated data from primary cancer samples in the experimental design. A strategy to combine and rank data from these different data sources is essential to minimize the experimental work in the validation steps.

**Aim:**

To apply a new relaxation ranking algorithm to enrich DNA methylation markers in cervical cancer.

**Results:**

The application of a new sorting methodology allowed us to sort high-throughput microarray data from both cervical cancer cell lines and primary cervical cancer samples. The performance of the sorting was analyzed *in silico*. Pathway and gene ontology analysis was performed on the top-selection and gives a strong indication that the ranking methodology is able to enrich towards genes that might be methylated. Terms like regulation of progression through cell cycle, positive regulation of programmed cell death as well as organ development and embryonic development are overrepresented. Combined with the highly enriched number of imprinted and X-chromosome located genes, and increased prevalence of known methylation markers selected from cervical (the highest-ranking known gene is *CCNA1*) as well as from other cancer types, the use of the ranking algorithm seems to be powerful in enriching towards methylated genes.

Verification of the DNA methylation state of the 10 highest-ranking genes revealed that 7/9 (78%) gene promoters showed DNA methylation in cervical carcinomas. Of these 7 genes, 3 (*SST*, *HTRA3 *and *NPTX1*) are not methylated in normal cervix tissue.

**Conclusion:**

The application of this new relaxation ranking methodology allowed us to significantly enrich towards methylation genes in cancer. This enrichment is both shown *in silico *and by experimental validation, and revealed novel methylation markers as proof-of-concept that might be useful in early cancer detection in cervical scrapings.

## Background

DNA methylation represents a modification of DNA by addition of a methyl group to a cytosine, also referred to as the fifth base [1]. This epigenetic change does not alter the primary DNA sequence and might contribute to overall genetic stability and maintenance of chromosomal integrity. Consequently, it facilitates the organization of the genome into active and inactive regions with respect to gene transcription [2]. Genes with CpG islands in the promoter region are generally unmethylated in normal tissues. Upon DNA hypermethylation, transcription of the affected genes may be blocked, resulting in gene silencing. In neoplasia, hypermethylation is now considered as one of the important mechanisms resulting in silencing expression of tumour suppressor genes, i.e. genes responsible for control of normal cell differentiation and/or inhibition of cell growth [3]. In many cancers, various markers have been reported to be hypermethylated [4]. The detection of DNA hypermethylation was revolutionized by two discoveries. Bisulfite treatment results in the conversion of cytosine residues into uracil, except the protected methylcytosine residues [5]. Based on the sequence differences after bisulfite treatment, methylated DNA can be distinguished from unmethylated DNA, using methylation specific PCR (MSP) [6].

In the last few years, hypermethylated biomarkers have been used in cancer research and diagnostics [7-9]. Presently, DNA hypermethylation of only few markers is of clinical relevance [9]. Two classical examples are hypermethylation of *MGMT *in the prediction of treatment response to temozolomide in glioblastoma [10] and DNA hypermethylation of GSTP1 in the early detection of prostate cancer [11]. The search for markers that are hypermethylated in specific cancer types resulted in a large list of genes but more recent evidence revealed that many of these markers are methylated in normal tissues as well [12,13].

To discover novel markers that are specific for certain stages of cancer with a high specificity and sensitivity, large-scale screening methods were developed such as Restriction Landmark Genomic Scanning [14], Differential Methylation Hybridization [15-17], Illumina GoldenGate^® ^Methylation, microarray-based Integrated Analysis of Methylation by Isoschizomers (MIAMI) [18] and MeDIP [19] in combination with methylation-specific oligonucleotide microarray [20]. These approaches demonstrated that large-scale screening techniques have a large potential to find novel methylation targets in a whole range of cancers. To identify cancer related hypermethylated genes, also pharmacological unmasking expression microarray approaches were suited [21-23]. In this approach, the re-activation of gene expression using microarray analysis was studied during functional reversal of DNA methylation and histone acetylation in cancer cell lines using demethylating agents and histone deacetylase inhibitors. This methodology generally results in a list of several hundreds of candidate genes. Although the analysis of the promoter (e.g. screening for dense CpG islands) is used to narrow down the number of candidate genes, the number list is still too large. This methodology has proven relevant as its application resulted in the identification of new potential methylated genes [24,25].

However, the initial large scale screening approach will also detect many genes that are not directly methylation targets themselves but become re-activated due to the re-expression of for instance transcription factors [26]. Furthermore, in most studies only re-expression data after demethylation in cell lines were used. Smiraglia and co-workers [27] calculated that more than 57% of the loci methylated in cell lines were never methylated in 114 primary cancers of different malignancy types. The small number of cell lines used to identify methylated genes does not allow to draw conclusions on the relevance of such cancer-specific genes without testing a large series of primary tumours, which is not done in most studies.

Finally, the completion of the sequence of the human genome provided information on genes, promoter gene structure, CG-content and chromosomal localization. These data are useful to define criteria for the candidate genes to act as appropriate targets for DNA methylation.

To identify genes that are downregulated due to promoter hypermethylation and to enrich for those genes that are most frequently involved in cervical cancer, we performed the following experiments:

• Affymetrix expression microarray analysis on a panel of frozen tissue samples from 39 human primary cervical cancers to identify cancer specific down-regulated genes

• To select those genes that are hypermethylated in cervical cancer, Affymetrix expression microarray analysis on a panel of 4 different cervical cancer cell lines in which the expression of (hyper)methylated genes was re-activated upon treatment with 5-aza-2'deoxycytidine (DAC) (blocking DNA methylation), and/or trichostatin A (TSA) (inhibiting histone deacetylase (HDACi))

• Data from both approaches were combined, and a novel non-parametrical ranking and selection method was applied to identify and rank candidate genes. Using *in silico *promoter analysis we restricted the analysis to those candidate genes that carry CpG islands

To validate whether our new approach resulted in a significant enrichment of hypermethylated genes, we compared the first 3000 high-ranking candidate probes with lists of imprinted genes, X-chromosome located genes and known methylation markers. In addition, to investigate whether the promoters of these selected gene probes are hypermethylated and this methylation is present in cancer and not in normal tissue, we determined the hypermethylation status of the 10 highest ranking candidate genes in both cervical cancers and normal cervices using COBRA (COmbined Bisulfite Restriction Analysis). These data revealed a highly significant enrichment of methylated genes.

## Methods

### Primary cervical tissue samples

For the expression microarray analysis, tissues from 39 early stage frozen cervical cancer samples were used from a collection of primary tumours surgically removed between 1993 and 2003. All patients were asked to participate in our study during their initial visit to the outpatient clinic of the University Medical Centre Groningen (UMCG, Groningen, The Netherlands). Gynaecological examination under general anaesthesia was performed in all cervical cancer patients for staging in accordance with the International Federation of Gynaecology and Obstetrics (FIGO) criteria [28]. Tumour samples were collected after surgery and stored at -80°C. The stage of cervical cancer patients included 33 FIGO stage IB (85%) and 6 FIGO stage IIA (15%). The median age of the cervical cancer patients was 46 years (IQ range 35 – 52 yr.).

For COBRA and BSP (Bisulfite Sequencing PCR), 10 (of the 39) primary cervical cancers and 5 controls (normal cervix) were used. The age-matched normal cervical controls were women without a history of abnormal Pap smears or any form of cancer and planned to undergo a hysterectomy for benign reasons during the same period. Normal cervices were collected after surgery and histologically confirmed.

Informed consent was obtained from all patients participating in this study. The study was approved by the ethics committee of the UMCG.

### Cervical cancer cell lines

Four cervical carcinoma cell lines were used: HeLa (cervical adenocarcinoma, HPV18), SiHa (cervical squamous cell carcinoma, HPV16), CSCC-7 (nonkeratinizing large cell cervical squamous cell carcinoma, HPV16) and CC-8 (cervical adenosquamous carcinoma, HPV45). HeLa and SiHa were obtained from the American Tissue Type Collection. CSCC-7 and CC-8 [29] were a kind gift of Prof. GJ Fleuren (Leiden University Medical Centre, Leiden, the Netherlands). All cell lines were cultured in DMEM/Ham's F12 supplemented with 10% fetal calf serum.

Cell lines were treated for 3 days with low to high dose (200 nM, 1 μM or 5 μM) 5-aza-2'deoxycytidine (DAC), 200 nM DAC with 300 nM trichostatin A (TSA) after 48 hours, or left untreated as described previously [21,23,30]. Cells were split to low density 24 hours before treatment. Every 24 hours DAC was refreshed. After 72 hours cells were collected for RNA isolation.

### RNA and DNA isolation

From the frozen biopsies, four 10-μm-thick sections were cut and used for standard RNA and DNA isolation. After cutting, a 3-μm-thick section was stained with haematoxylin/eosin for histological examination and only tissues with >80% tumour cells were included. Macrodissection was performed to enrich for epithelial cells in all normal cervices.

For DNA isolation, cells and tissue sections were dissolved in lysis buffer and incubated overnight at 55°C. DNA was extracted using standard salt-chloroform extraction and ethanol precipitation for high molecular DNA and dissolved in 250 μl TE-4 buffer (10 mM Tris; 1 mM EDTA (pH 8.0)). For quality control, genomic DNA was amplified in a multiplex PCR containing a control gene primer set resulting in products of 100, 200, 300, 400 and 600 bp according to the BIOMED-2 protocol [31].

RNA was isolated with TRIzol reagent (Invitrogen, Breda, the Netherlands) according to manufacturer's protocol. RNA was treated with DNAse and purified using the RNeasy mini-kit (Qiagen, Westburg, Leusden, the Netherlands). The quality and quantity of the RNA was determined by Agilent Lab-on-Chip analysis (ServiceXS, Leiden, the Netherlands, .

### Expression data

Gene expression for 39 primary cancers and 20 cell line samples (4 cell lines treated with various combinations of DAC/TSA) was performed using the Affymetrix HGU 133 Plus 2.0 array with 54,675 probes for analysis of over 47,000 human transcripts. The labelling of the RNA, the quality control, the microarray hybridization and scanning were performed by ServiceXS according to Affymetrix standards. For labelling, 10 μg of total RNA was amplified by in vitro transcription using T7 RNA polymerase.

Quality of the microarray data was checked using histograms, box plots and a RNA degradation plot. One cell line sample was omitted because of poor quality. Using BioConductor [32] present (P), absent (A) or marginal (M) calls were determined with the MAS5 algorithm (see Additional file 1 for data). MAS5 uses a non-parametric statistical test (Wilcoxon signed rank test) that assesses whether significantly more perfect matches show more hybridization signal than their corresponding mismatches to produce the detection call for each probe set [33]. The relaxation ranking approach only relied on P-calls. Some samples were analyzed in duplicate, and the profile of P-calls is highly similar (93–95% of the probesets have an identical P/M/A call).

### Relaxation ranking algorithm

In order to identify the most promising markers that are methylated in cervical cancer, we assumed that such markers should be silenced in cancer cells and upregulated upon re-activation after DAC/TSA treatment, Therefore, the ideal methylation markers will be genes represented by probes with:

• no expression in primary cervical cancers: P-calls = 0 out of 39 cancers

• no expression in (untreated) cervical cancer cell lines: P-calls = 0 out of 4 cell lines

• expression in cervical cancer cell lines treated with DAC (or DAC in combination with TSA): P-calls = 15 out of 15 treated cell lines

To select for those gene probes that would be the best candidate hypermethylated genes in cervical cancer, we present the relaxation ranking algorithm. Probesets were ranked, not primarily based on the number of P-calls and thus explicitly setting thresholds, but primarily driven by the number of probesets that would be picked up, based on selection criteria (the number of P-calls in primary cancers, untreated and treated cell lines). The stricter (e.g. P-calls: 0-0-15) these selection criteria, the lower the number of probes that meet with these criteria; while if the conditions become more and more relaxed (higher number of P-calls in primary cancers and untreated cell lines, and lower number of P-calls in treated cell lines), the more probes will comply. In the end, using P-calls: 39-4-0 as criteria, all probe sets were returned. This way, there was no need to define a 'prior' threshold for the number of P-calls.

The following sorting method was applied (R-scripts are presented in Additional file 2):

(1) All possible conditions were generated and the number of probes that were picked up under these conditions was calculated:

a. the number of samples with expression (P) of a certain probe in

i. primary cervical cancer samples is called x_sample_

ii. cervical cancer cell lines is called y_sample_

iii. treated cervical cancer cell lines is called z_sample_

b. all combinations of x, y and z are made

i. x (the number of P-calls in primary cancers) varies from 0 to 39

ii. y (the number of P-calls in untreated cell lines) from 0 to 4

iii. z (the number of P-calls in treated cell lines) from 0 to 15

iv. In total, 3200 combinations of x, y and z can be made

c. a probeset was found under each of these generated conditions x, y and z if:

i. x_sample _≤ x (number of P-calls for probe in primary cancers smaller or equal compared to condition) AND

ii. y_sample _≤ y (number of P-calls for probe in untreated cell lines smaller or equal compared to condition) AND

iii. z_sample _≥ z (number of P-calls for probe in treated cell lines larger or equal compared to condition)

d. under very strict conditions (x = 0, y = 0, z = 15) no probes were found, while under the most relaxed conditions (x = 39, y = 4, z = 0) all probes were returned. For all combinations of x, y and z, the number of probes that complied (w), was stored

(2) The data was sorted with w as primary criterion (ascending), followed by x (ascending), y (ascending) and z (descending)

(3) This sorted dataset was analyzed row per row. In row i, the w_i _probes retrieved with criteria x_i _y_i _z_i _were compared with the list of probes, already picked up in rows 1 to i-1. If a probe did not occur in this list, it was added to the list

(4) This process continued until there were m (user-defined) probes in the list

All *in silico *statistical enrichment tests are chi-square tests with Yates' correction, given p values are two-tailed.

### DNA methylation analysis using COBRA and bisulfite sequencing (BSP)

To validate the (hyper)methylation status of candidate gene probes, DNA extracted from 10 cervical cancers and 5 normal cervices were analyzed using BSP and COBRA. Bisulfite modification of genomic DNA was performed using the EZ DNA methylation kit (Zymogen, BaseClear, Leiden, the Netherlands). The 5' promoter region of the tested gene was amplified using bisulfite treated DNA. PCR primers for amplification of specific targets sequences are listed in Additional file 3. COBRA was performed directly on the BSP products as described by Xiong et al. [34]. using digestions with *BstUI*, *Taq1 *and/or *HinfI *according the manufacture's protocol (New England Biolabs Inc., Beverly, MA). For sequence analysis, the BSP products were purified (Qiagen, Westburg, Leusden, the Netherlands) and subjected to direct sequencing (BaseClear, Leiden, the Netherlands). Leukocyte DNA collected from anonymous healthy volunteers and in vitro CpG methylated DNA with *SssI *(CpG) methyltransferase (New England Biolabs Inc., Beverly, MA) were used as negative and positive control, respectively.

## Results

To identify novel markers that are methylated in cervical cancer, we applied a multistep approach that combines re-expression of silenced hypermethylated genes in cervical cancer cell lines (using DAC and DAC/TSA), downregulated expression in 39 cervical cancers expression, and selection of candidate markers using a relaxing ranking algorithm. The best profile of a candidate marker would be: no expression in any of the 39 cervical primary cancers and 4 untreated cancer cell lines, but re-activation of expression after demethylation and/or blocking of histone deacetylation in all 15 cell lines treated with various combinations of DAC/TSA (P-calls: 0-0-15). However, none of the probe sets showed this ideal profile. To generate a list of candidate genes, a relaxation ranking algorithm was applied.

**Figure 1:**
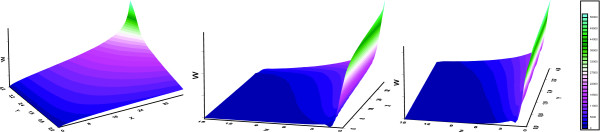
The number of probes (w) that is retrieved using parameters x (number of P-calls in primary cancers for probe), y (number of P-calls in untreated cell-lines for probe) and z (number of P-calls in treated cell-lines for probe).

The only variable used in the relaxation ranking is the number of probes we would like to retrieve. As shown in Figure 1, the number of probes retrieved (w) with parameters x, y and z (the number of P-calls in respectively primary tumour samples, untreated and treated cell lines) follows a complex profile which consists not only of additive elements, but also interactions between the parameters. In general, the number of P-calls in primary cancer samples (x) has the largest influence on w. The sorting methodology has the advantage that no cut-off values have to be chosen for x, y and z, and therefore there is no need to implicitly link a relative weight factor to the parameters.

**Figure 2:**
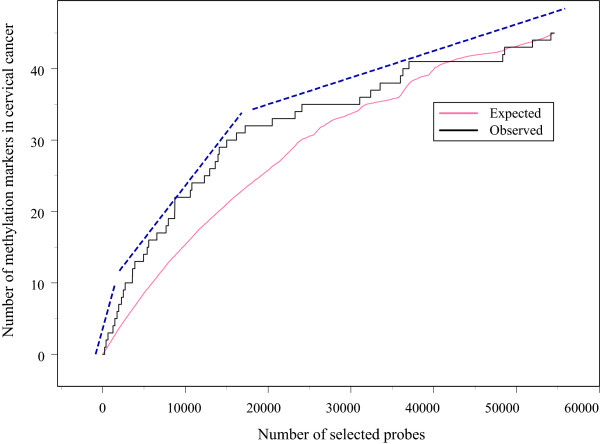
**Step-plot to determine optimal number of probes for further analysis**. Step-plot of the number of retrieved known markers (45 published hypermethylation markers in cervical cancer, see Additional file 4) as a function of the position after relaxation ranking (this is the number of selected probes after ranking). The step plot shows the actual (observed) number of markers. If the markers were randomly distributed, one would expect the profile, marked with 'expected' (details in the text). The trend of the observed markers versus the number of selected probes is indicated with dashed lines.

To calculate the most optimal number of potentially hypermethylated candidate markers for further analysis, we estimated this number based on known (i.e. described in literature) methylation markers in cervical cancer. Forty-five known methylation markers were found using text-mining using GeneCards [35] as source of aliases/symbols to query PubMed through NCBI E-Utils (Additional file 4). The position of the markers after ranking ("observed") was determined as shown in the step plot in Figure 2. If the markers would be randomly distributed in the ranking, the profile would be similar to the curve, marked 'expected'. This 'expected' curve is not a straight line, but is calculated based on whether a probe could be assigned with a gene symbol and taking probes into account that are associated with a gene that was already associated with an earlier selected probe. The number of observed methylation markers has in general the same slope as expected. However, up to about 3000 probes, the slope of the number observed markers versus the number of selected probes (in dashed lines) cannot be explained if the markers would be randomly distributed as its steepness is much higher. When selecting more than 3000 probes, the slope suddenly decreases to a level that is close to random distribution. This enrichment can also statistically be proven (see further). Therefore, we selected the first 3000 probes, referred to as TOP3000, in the ranking for further analysis. In this TOP3000 list, 2135 probes are associated with a gene symbol, of which 1904 are unique.

### The validation of the TOP3000 probe-list selected using relaxing high-ranking

To validate whether the TOP3000 contains potential hypermethylated genes, we determined the occurrence of various gene sets that are known to be hypermethylated such as imprinted genes, chromosome-X genes, cervical cancer related hypermethylated genes and genes reported to be methylated frequently in cancers, other than cervical cancer.

#### A. Enrichment for imprinted genes

Imprinting is a genetic mechanism by which genes are selectively expressed from the maternal or paternal homologue of a chromosome. As methylation is one of the regulatory mechanisms controlling the allele-specific expression of imprinted genes [36-40], it is expected that known imprinted genes are enriched in the TOP3000 selection. According to the Imprinted Gene Catalogue [41], this TOP3000 list contains 16 imprinted (or parent-specific expressed) genes (Additional file 5). On the whole Affymetrix array in total 74 imprinted genes could be assigned with a probe. Taking into account duplicate probes and probes that are not associated with a gene symbol, 8.76 imprinted genes could be expected in the first 3000 probes if the imprinted genes were randomly distributed indicating a 1.83-fold [16/8.76] enrichment in the TOP3000 (X^2 ^= 5.904; p = 0.0151). The enrichment towards imprinted genes is even more significant in the TOP100 candidate genes (3 versus only 0.31 expected; X^2 ^= 14.9; p < 0.0001).

#### B. Enrichment for genes on the X-chromosome

X-chromosome-inactivation in females is initiated from an inactivation centre that produces the *Xist *transcript, an RNA molecule that covers one copy of the X-chromosome and results in silencing of gene expression. This coating initiates a number of chromatin changes including stable DNA methylation [42]. Of the entire list of 54675 probes on this Affymetrix array, 40683 could be associated with a chromosomal location, 1325 probes are located on the X-chromosome. In the TOP3000 list (with 2239 chromosomal locations known), 93 probes are located on chromosome X (data not shown) indicating a significant enrichment (1.28-fold) of X-chromosome-located probes in the TOP3000 (X^2 ^= 5.8; p = 0.0165). This enrichment is even more significant within the TOP1000 (42/708 chromosomal locations; X^2 ^= 12.567; p = 0.0004) and TOP100 probes (13/71 chromosomal regions known; X^2 ^= 36.097; p < 0.0001).

#### C. Enrichment for cervical cancer specific methylation markers

The enrichment of known methylation markers involved in cervical cancer (Additional file 4) was already illustrated significant when calculating the optimal number of probes for further testing (see Figure 2), and hereby demonstrated the enrichment towards these markers. In the TOP3000, 10 known genes are present (Table 1). As only 5.33 probes of these known methylation markers for cervical cancer are expected if randomly distributed, the TOP3000 list is enriched for these markers 1.88-fold (X^2 ^= 3.715 ; p = 0.0539).

**Table 1:** Reported DNA methylation markers in cervical cancer present in the TOP3000

**Gene symbol**	**Rank**	**Profile X-Y-Z**	**Chromosomal location**	**References**
*CCNA1*	234	3-0-7	13q12.3-q13	[48]
*TIMP2*	404	20-3-14	17q25	[62]
*TFPI2*	651	12-0-7	7q22	[22]
*PEG3*	1242	10-0-6	19q13.4	[63]
*RUNX3*	1463	39-3-15	1p36	[64]
*IGSF4*	1742	38-0-7	11q23.2	[65]
*PTEN*	1926	15-1-8	10q23.3	[66]
*TNFRSF10D*	2270	30-4-15	8p21	[67]
*TIMP3*	2500	32-3-13	22q12.3	[13]
*APC*	2733	11-0-5	5q21-q22	[13]

Published DNA methylation markers in cervical cancer were selected by literature text mining (see Additional file 4)

#### D. Enrichment for known hypermethylation markers in cancers other than cervical cancer

To determine whether the ranking methodology is able to enrich towards known hypermethylation markers reported in various cancer types, PubMeth (a literature-based methylation database) was used [43]. Of the 40683 gene-probes on the Affymetrix array, 349 genes are present in the database. Interestingly, in the TOP250 probes (representing 152 unique genes), 10 known methylation are described in the database (see table 2). If randomly distributed, taking duplicate probes and probes not associated with gene symbols into account, 3.3 genes were expected (X^2 ^= 12.028, p = 0.0005). This enrichment is also observed in the TOP1000 (27 known markers vs. 14 expected, X^2 ^= 11.947, p = 0.0005) and TOP3000 probes (55 known markers vs. 41 expected, X^2 ^= 4.871, p = 0.0273).

**Table 2:** Listing of cancer associated hypermethylation markers that have been reported previously within the 250 highest ranking genes, as found by literature search (trough NCBI E-fetch, using GeneCards to search aliases)

**Gene symbol**	**Rank**	**Profile X-Y-Z**	**Chromosomal location**	**References**
*ZIK1*	6	16-1-15	19q13.43	Intestinal metaplasia [68]
*NNAT *^1,2^	21	0-1-11	20q11.2-q12	Pediatric acute leukemia [40]
*SST*^1^	22	1-1-12	3q28	Colon cancer [53]
*SSX2*^3^	27	0-0-8	Xp11.23-p11.22	Bladder cancer [69]
*NPTX1*^1^	29	2-3-14	17q25.1-q25.2	Pancreatic cancer [54]
*PRSS21*	72	18-3-15	16p13.3	Testicular cancer [70,71]
*CYP1A1*^1^	76	0-0-7	15q22-q24	Prostate cancer [72]
*MAGEA3*^1,3^	96	4-2-12	Xq28	Different cancer cell lines (Leukemic, Hepatic, Prostate, Breast, Colon) [73] Hepatocellular carcinoma [74] Melanoma [75]
*INSR*	124	7-0-9	19p13.3-p13.2	Prostate cancer [50]
*DLX1*	150	2-3-12	2q32	Lung cancer [76]
*PAX9*	207	25-1-12	14q12-q13	Lung cancer [76]
*ZNF342*	228	0-0-6	19q13.32	Brain cancer [77]
*CCNA1*^1,4^	234	3-0-7	13q12.3-q13	Cervical cancer [48] Head-and neck cancer [30] Oral cancer [52]
*CTCFL*	245	14-0-9	20q13.31	Prostate and bladder cancer [78]
*LIFR*	248	32-3-15	5p13-p12	Hepatocellular carcinoma [79]

^1^Genes, whose promoter has been described in literature as being methylated in certain cancer types selected by screening the PubMeth database. ^2^Imprinted. ^3^Located on the X-chromosome. ^4^Methylated in cervical cancer (literature search)

Interestingly, this analysis revealed that in the TOP-250 known methylation markers seem to be significantly enriched and highly-ranked. This also showed that the known cervical cancer-specific markers are not enriched to the same extend (*CCNA1 *is highest at position 234), implying the existence of better hypermethylated markers, involved in cervical cancer, in the TOP3000 list.

In summary, as the top-list contains a relatively large number of imprinted, chromosome-X and known methylation markers, our analysis revealed that the ranking strategy was able to enrich the candidate gene list with possible new (hyper)methylated genes.

#### E. Gene Ontology

Associated with the development and progression of cancer, silencing by hypermethylation often affects genes in important pathways [44]. Therefore, we investigated whether our selected TOP3000 candidate genes are associated with specific pathways or harbour related functions. Multiple GO-terms using Gene Ontology (GO) by GOstat [45] (Additional file 6) and specific pathways using Ingenuity Pathway Analysis (IPA) (Additional file 7), were significantly over-represented within the TOP3000 list when compared to all annotated human genes.

These terms include regulation of transcription – DNA-dependent, transcription from RNA polymerase II promoter, regulation of progression through cell cycle, positive regulation of programmed cell death as well as organ development and embryonic development (all p-values < 10^-6 ^; corrected for multiple hypothesis testing [46]).

Genes in these processes were reported to be often methylated during cancer progression [8]. Genes responsible for development and differentiation are mainly silenced by methylation in normal tissues. On the other hand, in cancer tissues, genes responsible for cell cycle control and induction of apoptosis are often aberrantly expressed as many of these genes have tumour suppressor activity. DNA hypermethylation is one mechanism to regulate expression of tumour suppressor genes [47].

The GO-analysis provided additional strong indication that our highest ranking genes in the top-list are significantly enriched for methylated genes involved in cervical cancer tissue or cell lines.

### Validation of the 10 highest-ranking candidate genes using COBRA

In order to validate whether the highest ranking genes represent markers that are functionally hypermethylated in cervical cancer, we performed COBRA on bisulfite treated DNA of 10 cervical cancers and 5 normal cervices. For this analysis, we focused on those first 10 genes from the highest ranking probe-list (Table 3) that (see Additional file 8 for more details):

**Table 3:** Methylation status using COBRA of the 10 highest-ranking gene promoters.

**Rank**	**Gene symbol**	**Profile X-Y-Z**	**Chromosomal location**	**Methylation in cancer**	**Methylation in normal**
1	*DAZL*	1-1-13	3p24.3	9/9	5/5
2	*ADARB1*	1-2-15	21q22.3	Nd	Nd
3	*SYCP3*	0-1-12	12q	9/9	5/5
4	*AUTS2*	12-0-12	7q11.22	0/9	0/5
5	*NNAT*	0-1-11	20q11.2	9/9	5/5
6	*SST*	1-1-12	3q28	7/9	0/5
7	*HTRA3*	6-0-10	4p16.1	1/9	0/5
8	*ZFP42*	11-1-14	4q35.2	9/9	5/5
9	*NPTX1*	2-3-14	17q25.1	5/10	0/5
10	*GDA*	14-3-15	9q21.13	0/9	0/5

47	*CCNA1*	3-0-7	13q12.3-q13	6/10	0/5

**Figure 3:**
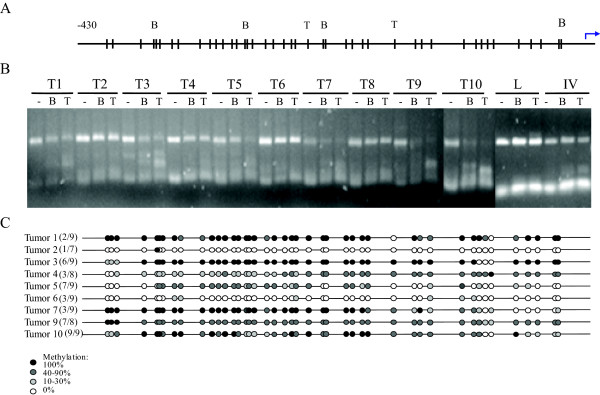
**(Hyper)methylation analysis of the promoter region (-430 to -5 of TSS) of the *CCNA1 *gene by COBRA and sequence analysis.** A: schematic representation of the restriction enzyme sites (B: *BstUI *and T: *TaqI*) in the virtual hypermethylated BSP nucleotide sequence after bisulfite treatment. Vertical bars represent CG site, arrow represents TSS (retrieved from Ensembl). B: Result of COBRA analysis of the BSP products of 10 tumour samples (T1-T10), in vitro methylated DNA as a positive control (IV) and leucocyte DNA as a negative (unmethylated) control (L). C. Schematic representation of the sequencing results. From each tumour, the BSP-products were cloned into TOPO-pCR4 (Invitrogen) and sequencing (BaseClear) was performed on M13-PCR products of 7–9 independent clones. Circles represent CG dinucleotides: the darker, the more clones at this site were methylated.

**Figure 4:**
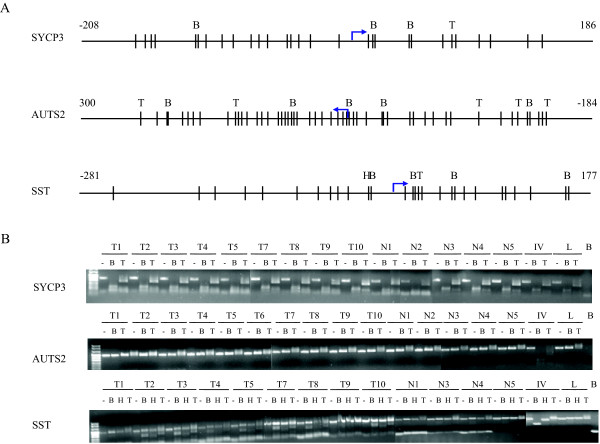
**Representative COBRA on 3 gene promoters (*SST*, *AUTS2 *and *SYCP3*)**. A: schematic representation of of the restriction enzyme sites in the virtual hypermethylated BSP nucleotide sequence after bisulfite treatment.(B: *BstUI*, T: *TaqI *and H: *HinfI*). Bars represent CG site and arrow is TSS (retrieved from Ensembl). B: Result of COBRA analysis of BSP products of tumour samples (T1-T10) and 5 normal cervices (N1-N5), in vitro methylated DNA as a positive control (IV) and leukocyte DNA as a negative (unmethylated) control (L); lane B is water blank.

Gene, selected for further validation after applying additional criteria. Included is CCNA1 on position 47 (original position 241) as the highest ranking cervical-cancer-associated hypermethylated gene (see **Table 2**). Methylation status was determined by BSP/COBRA (see Figure 3 and Figure 4).

• represent a known gene (i.e. gene symbol)

• contain a CpG island surrounding the TSS

• are located on any chromosome except chromosome X

• are expressed (present) in less than 15 carcinomas, in order to identify markers to be methylated in ≥60% of cervical cancers (no P-call)

BSP was used to amplify the CpG islands of these candidate genes using bisulfite-treated DNA and COBRA to determine the methylation status. *CCNA1 *(at position 49; Additional file 8) was included as a positive control for the highest listed, reported cervical cancer specific methylation gene promoter. COBRA of *CCNA1 *revealed that 6 of 10 carcinomas are methylated at the restriction enzyme sites (T1, T3, T5, T7, T9 and T10 in Figure 3). Sequence analysis of the BSP products (on average 7–9 independent clones for each carcinoma) of these 10 carcinomas revealed that in 6 carcinomas the promoter is hypermethylated in good agreement with the COBRA results (Figure 3C).

Table 3 summarizes the methylation status of the 10 highest ranking genes in 10 cervical cancer and 5 normal cervices using COBRA. One gene (*ADARB1 *at rank 2) could not be analyzed for methylation as no specific BSP products could be amplified using several combinations of primer pairs. Interestingly, using the BSP products of the other 9 listed genes, 7 (78%) showed methylation in carcinomas (Table 3).

Four genes are hypermethylated in all 9 tested cancers, while for *SST *(7 of 9 carcinomas), *HTRA3 *(1 of 9 carcinomas) and *NPTX1 *(5 of 10 carcinomas) a fraction of the tested carcinomas is hypermethylated. Figure 4 shows a representative methylation analysis of 3 genes using COBRA. Three (*NNAT*, *SST *and *NPTX1*) of the 7 hypermethylated gene promoters have been reported to be methylated in tumours previously (see Table 2). Taken these data together, these findings showed that the relaxation ranking algorithm resulted in a very significant enrichment towards genes with a positive methylation status.

### Enrichment of cervical cancer specific methylation markers

A cancer specific cervix hypermethylation marker is only of relevance for the diagnosis of (pre-) malignant disease in case normal cervical epithelium is not methylated. COBRA analysis of 5 normal cervices for all 9 genes revealed that 4 genes (*DAZL*,*SYCP3*,*ZFP42 *and *NNAT*) are hypermethylated in all 5 samples (Table 3).

On the other hand, of the 7 genes hypermethylated in cervical cancer specimens, 3 genes (*SST*, *HTRA3 *and *NPTX1*) did not show DNA methylation in any of the normal cervices of 5 independent individuals. We observed the same methylation profile for *CCNA1 *that was reported previously as a cervical cancer specific gene [48] with hypermethylation in only 6 of 10 tumours but none of the 5 normals (Table 3). This analysis revealed that the relaxation ranking algorithm not only resulted in a very significant enrichment for genes with a positive methylation status, but also for hypermethylated genes that are specifically methylated in cancers and not in the normal cervices.

## Discussion and conclusion

In this study, we optimized the identification of methylation markers after pharmacological unmasking microarray approach combined with microarray expression data of primary cancer samples. For the integration of data from both cell lines and primary cancers, we developed a novel ranking strategy, which combines re-activation in cell lines and no expression in primary cancer tissue. The relaxation ranking algorithm uses a non-parametrical method of sorting. No threshold on expression level or P-calls has to be set and no overlap between different cell lines has to be chosen. The only parameter needed is the number of probes/genes that should be included in the top list. Using this algorithm, genes can still be selected for further analysis, even if it is not in (almost) all cell lines re-expressed or not silenced in most primary tumour samples.

In this study, we showed that the experimental design in combination with the ranking strategy is able to enrich a list of probes for methylated genes. Imprinted genes and genes on the X-chromosome are significantly enriched in the high-ranking TOP3000 probes. Pathway and gene ontology analysis illustrates that the high-ranking genes are involved in tumour development and progression. Enrichment of similar pathways or ontologies when selecting abnormal expressed genes is commonly reported in various cancer types [49,50]. More importantly, methylation markers reported to be involved in various cancers (including cervical cancer) are significantly enriched in the top-lists as well. Interestingly, the highest ranking cervical cancer specific gene is *CCNA1 *(position 234 in Additional file 8; position 47 in Table 3). Apart from cervical cancer, *CCNA1 *was reported to be hypermethylated in colorectal, oral and head and neck cancer [30,51,52]. In good agreement with the reported data, we show that *CCNA1 *is hypermethylated in 6 of 10 cervical carcinomas and none of the normal cervices using COBRA and BSP sequencing (Table 3 and Figure 3).

Analysis of the methylation status of the highest ranking genes revealed that seven out of nine selected genes (78%) are methylated in cervical cancers, whereas 4 of these 7 genes (*DAZL*, *SYCP3*, *ZFP42 *and *NNAT*) were also hypermethylated in all 5 normal cervices (Table 3). Although hypermethylation of *NNAT *has been implicated in paediatric acute leukaemia [40], the hypermethylation status in both cancer and normal tissues suggests that *NNAT *acts as an imprinted gene (Additional file 5) rather than a cancer specific methylation marker in cervical cancer. The other three genes (*SST*, *HTRA3 *and *NPTX1*) might be cancer specific because these genes are, similar as *CCNA1*, hypermethylated in the cancers and not in the normal controls (Table 3). Of these genes, two were previously described as cancer specific genes: *SST *in colon carcinoma [53] and *NPTX1 *in pancreatic cancer [54]. However, all 3 genes have not been described previously in cervical cancer. The exact involvement in cervical cancer development of these 3 genes has to be explored in the future, but the application of the relaxation ranking algorithm illustrates the power of enrichment for new hypermethylated genes that can discriminate between cervical cancer and normal cervical epithelium.

The combination of the initial setup and the analysis is unique. In most other studies either few genes are investigated for their methylation status in primary cancer samples or a large-screening approach is applied on cell lines only. Generally, only genes which are re-expressed in most cell lines can be retained for further investigation, as several hundreds of genes are upregulated in one or more cell lines after treatment with DAC/TSA. Most studies used additional filtering (such as pathway analysis, known mutated genes), but the list of candidate genes that need experimental validation to determine their methylation status is long. These markers need to go through a pipeline of DNA methylation detection in cell lines and cancer samples, in order to find only a few cancer specific markers with different sensitivity and specificity [21,23,53]. However, the success rate is relatively low, as many promoter regions do not show (differential) methylation. In addition, CpG arrays can be used to identify putative methylation markers, as recently described for cervical cancer [55]. Again, this method requires the analysis of many markers to end up with only few cervical cancer specific methylation markers.

In the last few years it became apparent that many markers that are methylated in cancer have been shown to be methylated in normal tissues as well [12,13,56,57]. Our present analysis once more illustrates that many more genes, preceded by a CpG island in the promoter region, are methylated in normal tissue as well than was previously anticipated. To be able to further increase the enrichment for these cancer specific methylated markers, the inclusion of expression microarray data from normal tissue in the relaxation ranking algorithm analysis might be helpful. To validate this, we performed global gene expression microarray analysis using the Affymetrix HGU 133 Plus 2.0 array with 54,675 probes (in parallel with the samples described in this study) on 5 independent age-matched normal cervices from healthy women. We assume that cancer specific methylated markers should be expressed in all normal cervices resulting in a positive P-call (most optimal P-call = 5). Including the P-call for normal expression on our 10 highest ranked methylated genes and *CCNA1 *(as listed in Table 3), revealed that all the four cervical cancer specific methylated genes (*SST*, *HTRA3*, *NPTX1 *and *CCNA1*) would not have been selected as none of the normal cervices showed a P-call for these probes (data not shown). It is generally accepted that tumor suppressor genes (including cancer specific methylated genes) are characterized by the fact that their expression can be downregulated as the result of methylation, mutations and/or deletions, but is still present in its normal counterpart tissue. However, the expression levels in normal tissue are relatively low for most of these genes when compared to those cancer tissues that do not show downregulation as was reported for p16^INK4a ^[58]. Thus, our data suggest that the addition of expression data of normal cervices would not enrich for cervical cancer specific methylated genes.

Other possibilities to further refine the selection of cancer relevant hypermethylated genes are to restrict the ranking to gene promoters that are likely to be methylated because of defined CG-content or the presence of conserved motifs (or similar sequence attributes) related to hypermethylated promoters [59-61]. Recently, we identified novel methylation markers, based on a genome-wide promoter alignment [57]. Promoters, closely related in the alignment with known methylation markers show to have a high chance to be methylated as well.

In conclusion, the application of this new relaxation ranking methodology allowed us to significantly enrich towards methylation genes in cancer. This enrichment is both shown *in silico *and by experimental validation, and revealed novel methylation markers as proof-of-concept that might be useful in early cancer detection in cervical scrapings.

## Competing interests

GBAW and AJK have been funded by OncoMethylome Sciences SA, Liège, Belgium. AGJvdZ is a paid consultant of OncoMethylome Sciences SA, Liège, Belgium and WvC is an employee of OncoMethylome Sciences SA, Liège, Belgium.

## Authors' contributions

MO did all the statistical analysis and wrote the manuscript. GBAW contributed to the microarray experiments and methylation analysis, wrote the manuscript and supervised the study. HHV performed the COBRA experiments. AJK contributed to the microarray experiments and performed BSP analysis. AGJvdZ initiated and supervised the study. WvC initiated the relaxation algorithm and supervised the statistical part of the study. ES wrote the manuscript and supervised the study. All authors read and approved the manuscript.

## Pre-publication history

The pre-publication history for this paper can be accessed here:



## Supplementary Material

Additional file 1 **Supplementary file 1**. Microarray analysis data determined by MAS5 algorithm for all samples, cell lines -/+ treatment and cervical cancers, represented by present (P), marginal (M) or absent (A). Click here for file

Additional file 2 **Supplementary file 2**. R-scripts of relaxation ranking algorithm. Click here for file

Additional file 3 **Supplementary table 1**. list of primers used for BSP. Click here for file

Additional file 4 **Supplementary table 2**. Overview of the 45 known methylation markers in cervical cancer selected from literature search and their position after relaxation ranking. Click here for file

Additional file 5 **Supplementary table 3**. Overview of published imprinted genes (Imprinted Gene Catalog), their position and gene name after relaxation ranking. Click here for file

Additional file 6 **Supplementary table 4**. enriched gene ontology terms, descriptions, number of genes associated with this GO term and P-value versus all human genes in the first 3000 probes. GO terms and statistics as determined by GOStat. Click here for file

Additional file 7 **Supplementary table 5**. Overview of Ingenuity networks, highly represented in the top-3000 list. Click here for file

Additional file 8 **Supplementary table 6**. The ranking of possibly functional methylated genes from the highest-ranking probe-list (TOP250). Probes were ranked according the relaxation ranking algorithm ("original ranking"). Possible functionally methylated genes were selected ("new ranking") by omitting probes that do not fulfill the additional criteria. Click here for file
