# Supplementary material for: Discovery of DNA methylation markers in cervical cancer using relaxation ranking
Source: BMC Med Genomics. 2008 Nov 24;1:57. doi: 10.1186/1755-8794-1-57 (PMC2605750; doi:10.1186/1755-8794-1-57)
Supplement: Additional file 2 — Supplementary file 2. R-scripts of relaxation ranking algorithm. [file 1755-8794-1-57-S2.doc]

# 0BSupplementary material

## 79BMaterials and methods

**R-scripts of relaxation ranking algorithm**

#Results-> affy probe ID in column 1; P-calls for primary cancers (0-39, in column 2); for cell-lines (0-4, in column 3); for treated cell-lines (0-15 in column 4)

count<-function(x,y,z){l<-length(results[results$V2<=x&results$V3<=y&results$V4>=z,1]);}

#count:function that counts how many rows in results are found with a certain condition

f<-matrix(nrow=3200,ncol=4)

#initialisation of f

i<-0;for (x in 0:39) {for (y in 0:4) { for (z in 0:15) {i<-i+1;f[i,1]<-count(x,y,z);f[i,2]=x;f[i,3]=y;f[i,4]=z; }}}

#simulate all conditions and store -> first column: number of genes (w), 2nd: x - 3th: y - 4th:z

ordered<-f[order(f[,1],f[,2],f[,3],-f[,4]),]

#order, first by w, followed by x, y (ascending) and z (descending)

kiezen<-function(x) {if (length(grep(x,list))>0){}else{list<<-append(list,x);}}

#function to find out whether a gene is allready in the list, if not, add it to the list

list<-"";counter<-1;n<-0;while(n<=3000) {counter<-counter+1;x2<-ordered[counter,2];y2<-ordered[counter,3];z2<-ordered[counter,4];temp<-results[(results$V2<=x2)&(results$V3<=y2)&(results$V4>=z2),1];found<-length(temp);counter2<-1;while (counter2<= found) {kiezen(as.character(temp[counter2]));counter2<-counter2+1;n<<-length(list);}}

#generate a list of n genes

#row per row, get x2, y2 and z2 from ordered

#check which genes are found with these criteria

#loop over these genes (names in first column of results)

#for each gene, check if already selected in previous round, if not, add to the list

#do this for more rows, until n genes reached ->these are in list
