# Supplementary material for: Discovery of DNA methylation markers in cervical cancer using relaxation ranking
Source: BMC Med Genomics. 2008 Nov 24;1:57. doi: 10.1186/1755-8794-1-57 (PMC2605750; doi:10.1186/1755-8794-1-57)
Supplement: Additional file 3 — Supplementary table 1. list of primers used for BSP. [file 1755-8794-1-57-S3.doc]

Supplementary table 1: list of primers used for BSP

| 1BName | 2BForward primer (5’-3’) | 3BReverse primer (5’-3’) | 4BTa | 5BStart position^1^ | 6BEnd position | 7BRefSeq |
| --- | --- | --- | --- | --- | --- | --- |
| DAZL | 8BTTTGGGGGTGATGTGTGTGTTT | 9BTCTCCCTCAACTCACCATAATA | 10B54 | 11B-161 | 12B312 | NM_001351 |
| ADARB1^2^ |  |  |  |  |  | 13BNM_015834 |
| SYCP3 | AAAATTTAAAAATTGGAAGGTATT AGG | ACCTCACTAATCAAAAACAACCTCT | 14B54 | 15B-208 | 16B+186 | NM_153694 |
| AUTS2 | 17BTTTTAAAAGTGATAAAGTTGGTTA TGG T | 18BCCCTTTTCTTTCTCCTCTCTTTCT | 19B56 | 20B+300 | 21B-184 | NM_015570 |
| NNAT | 22BGGTTAGGGATTGGGGAGAA | 23BGCTAAACTTACCTACAACAACAC | 24B54 | 25B-271 | 26B210 | NM_005386 |
| SST | 27BGGGGTATGTGGAATTGTGTG | 28BAAA TCT CCT TAC CTA CTT CCC C | 29B54 | 30B-185 | 31B+276 | NM_001048 |
| HTRA3 | 32BGTYGGTTTTGTYGTTATGTAGGY | 33BAAC TTC ACT TCC TCC CTA ACC | 34B57 | 35B+190 | 36B+622 | NM_053044 |
| ZFP42 | AGTAGGTGTTTGTTGAAGATAG | ACT CAT AAC ACA CAT AAC CAT C | 37B60 | 38B+308 | 39B+580 | NM_174900 |
| NPTX1 | 40BGGTAGTGGGGGTTTGATAG | 41BAAATAATCTCCTTCTACTACAACAC | 42B54 | 43B-2 | 44B+372 | NM_002522 |
| GDA | 45BTATAGAAGGTGGAGGAAGTTGA | 46BCACCTCCATAAAACAAATCCAAA | 47B54 | 48B-239 | 49B+194 | NM_004293 |
| CCNA1 | 50BTATAGTTGGAGTTGGAGGGT | 51BAAACAACTAACAAATACACTAAAA | 52B54 | 53B-279 | 54B+146 | NM_153694 |

^1^: +1 is transcription start site (TSS) ; ^2^: Several primer pairs were tested, however, none worked.
