# Supplementary material for: Discovery of DNA methylation markers in cervical cancer using relaxation ranking
Source: BMC Med Genomics. 2008 Nov 24;1:57. doi: 10.1186/1755-8794-1-57 (PMC2605750; doi:10.1186/1755-8794-1-57)
Supplement: Additional file 4 — Supplementary table 2. Overview of the 45 known methylation markers in cervical cancer selected from literature search and their position after relaxation ranking. [file 1755-8794-1-57-S4.doc]

Supplementary table 1: Overview of the 45 known methylation markers in cervical cancer selected from literature search and their position after relaxation ranking

| 55BRank | 56BProbe ID | 57BGene Name |
| --- | --- | --- |
| 234 | 205899_at | CCNA1 |
| 404 | 203167_at | TIMP2 |
| 651 | 209278_s_at | TFPI2 |
| 1242 | 209243_s_at | PEG3 |
| 1463 | 204197_s_at | RUNX3 |
| 1742 | 209032_s_at | IGSF4 |
| 1926 | 204054_at | PTENP1 |
| 2270 | 227345_at | TNFRSF10D |
| 2500 | 201147_s_at | TIMP3 |
| 2733 | 216933_x_at | APC |
| 3589 | 226389_s_at | RAPGEF1 |
| 3609 | 200824_at | GSTP1 |
| 3896 | 220804_s_at | TP73 |
| 4942 | 217561_at | CALCA |
| 5388 | 203139_at | DAPK1 |
| 5566 | 219550_at | ROBO3 |
| 6546 | 202037_s_at | SFRP1 |
| 7654 | 222713_s_at | FANCF |
| 7937 | 200665_s_at | SPARC |
| 8699 | 1558797_at | ROBO1 |
| 8710 | 209897_s_at | SLIT2 |
| 8734 | 215551_at | ESR1 |
| 10590 | 1555271_a_at | TERT |
| 10772 | 204346_s_at | RASSF1 |
| 12291 | 1562857_at | CDH13 |
| 12935 | 201130_s_at | CDH1 |
| 13595 | 211851_x_at | BRCA1 |
| 13943 | 1566484_at | FHIT |
| 14073 | 240776_at | PGR |
| 14988 | 204803_s_at | RRAD |
| 16174 | 207109_at | POU2F3 |
| 17208 | 205080_at | RARB |
| 20493 | 204880_at | MGMT |
| 23251 | 204745_x_at | MT1G |
| 24079 | 209644_x_at | CDKN2A |
| 31063 | 1552925_at | PCDH10 |
| 32394 | 1557615_a_at | SLIT1 |
| 33526 | 1561574_at | SLIT3 |
| 35987 | 205714_s_at | ZMYND10 |
| 36278 | 206657_s_at | MYOD1 |
| 37007 | 208461_at | HIC1 |
| 48347 | 204121_at | GADD45G |
| 48574 | 211163_s_at | TNFRSF10C |
| 51948 | 230378_at | SCGB3A1 |
| 54166 | 206983_at | FGFR1OP |

Genes selected using Textmining through NCBI E-Fetch, using GeneCards for aliases (www.genecards.org)
