# Supplementary material for: Discovery of DNA methylation markers in cervical cancer using relaxation ranking
Source: BMC Med Genomics. 2008 Nov 24;1:57. doi: 10.1186/1755-8794-1-57 (PMC2605750; doi:10.1186/1755-8794-1-57)
Supplement: Additional file 5 — Supplementary table 3. Overview of published imprinted genes (Imprinted Gene Catalog), their position and gene name after relaxation ranking. [file 1755-8794-1-57-S5.doc]

Supplementary table 1: Overview of published imprinted genes (Imprinted Gene Catalog), their position and gene name after relaxation ranking

| 58BRank | 59BImprinted rank | 60BGene Name |  |  |
| --- | --- | --- | --- | --- |
| 21 | 1 | NNAT (Evans et al., 2001) | | |
| 49 | 2 | H19  (Zhang et al., 2006; Rachmilewitz et al., 1992; Zhang and Tycko, 1992) | | |
| 59 | 3 | HFE (Bulaj et al., 1996) | | |
| 118 | 4 | MEG3 (Miyoshi et al., 2000) | | |
| 355 | 5 | NF2 (Evans et al., 1992) | | |
| 703 | 6 | ZIM2 (Murphy et al., 2001) | | |
| 769 | 7 | PPP1R9A (Nakabayashi et al., 2004) | | |
| 818 | 8 | PON2 (Ono et al., 2003) | | |
| 1241 | 9 | PEG3 (Murphy et al., 2001) | | |
| 1246 | 10 | CPA4 (Bentley et al., 2003) | | |
| 1605 | 11 | GABRA5 (Meguro et al., 1997) | | |
| 1951 | 12 | PEG10 (Ono et al., 2003) | | |
| 2001 | 13 | L3MBTL (Li et al., 2004) | | |
| 2788 | 14 | PHLDA2 (Lee and Feinberg, 1998) | | |
| 2855 | 15 | CDKN1C (Taniguchi et al., 1997) | | |
| 2948 | 16 | GNAL (Corradi et al., 2005) | | |
| 3896 | 17 | TP73 | | |
| 4623 | 18 | SLC38A4 | | |
| 4635 | 19 | HTR2A | | |
| 4674 | 20 | IL4R | | |
| 4843 | 21 | SGCE | | |
| 6591 | 22 | DIRAS3 | | |
| 6938 | 23 | PON3 | | |
| 7195 | 24 | ASCL2 | | |
| 7877 | 25 | GABRG3 | | |
| 8396 | 26 | ZNF215 | | |
| 8722 | 27 | PON1 | | |
| 8901 | 28 | SPINK5 | | |
| 8943 | 29 | NDN | | |
| 9497 | 30 | OSBPL5 | | |
| 9843 | 31 | PHF11 | | |
| 10226 | 32 | ASB4 | | |
| 10805 | 33 | GRB10 | | |
| 11994 | 34 | IGF2R | | |
| 13384 | 35 | TSPAN32 | | |
| 13891 | 36 | DCN | | |
| 13909 | 37 | CTNNA3 | | |
| 13960 | 38 | KCNQ1 | | |
| 14256 | 39 | ZNF264 | | |
| 14331 | 40 | SLC22A18 | | |
| 14423 | 41 | TSSC4 | | |
| 14943 | 42 | C6orf66 | | |
| 16963 | 43 | DLX5 | | |
| 17595 | 44 | IGF2 | | |
| 19031 | 45 | DIO3 | | |
| 19245 | 46 | NAP1L5 | | |
| 19840 | 47 | WT1 | | |
| 20712 | 48 | GABRB3 | | |
| 21448 | 49 | APP | | |
| 21500 | 50 | CD81 | | |
| 21677 | 51 | M6PR | | |
| 22329 | 52 | MEST | | |
| 22333 | 53 | SDHD | | |
| 23653 | 54 | BRD2 | | |
| 24342 | 55 | UBE3A | | |
| 29362 | 56 | PLAGL1 | | |
| 31040 | 57 | TCEB3C | | |
| 31095 | 58 | ZIM3 | | |
| 35914 | 59 | DDC | | |
| 36087 | 60 | SLC22A18AS | | |
| 36250 | 61 | MKRN3 | | |
| 36255 | 62 | INS | | |
| 36766 | 63 | CALCR | | |
| 36894 | 64 | MAS1 | | |
| 37122 | 65 | DLK1 | | |
| 37350 | 66 | RASGRF1 | | |
| 38039 | 67 | ATP10A | | |
| 39762 | 68 | IGF2AS | | |
| 39787 | 69 | KCNQ1DN | | |
| 39902 | 70 | USP29 | | |
| 40591 | 71 | TRPM5 | | |
| 42765 | 72 | PWCR1 | | |
| 51829 | 73 | GNGT1 | | |
| 52756 | 74 | MS4A2 | | |

Genes were selected using the Imprinted Gene Catalogue (Morison et al., 2005)

# 78BReferences

Bentley,L. et al. (2003) The imprinted region on human chromosome 7q32 extends to the carboxypeptidase A gene cluster: an imprinted candidate for Silver-Russell syndrome. *Journal of Medical Genetics*, 40, 249-256.

Bulaj,Z.J. et al. (1996) Clinical and biochemical abnormalities in people heterozygous for hemochromatosis. *New England Journal of Medicine*, 335, 1799-1805.

Corradi,J.P. et al. (2005) Alternative transcripts and evidence of imprinting of GNAL on 18p11.2. *Molecular Psychiatry*, 10, 1017-1025.

Evans,D.G.R. et al. (1992) A Genetic-Study of Type-2 Neurofibromatosis in the United-Kingdom .1. Prevalence, Mutation-Rate, Fitness, and Confirmation of Maternal Transmission Effect on Severity. *Journal of Medical Genetics*, 29, 841-846.

Evans,H.K. et al. (2001) The neuronatin gene resides in a "micro-imprinted" domain on human chromosome 20q11.2. *Genomics*, 77, 99-104.

Lee,M.P. and Feinberg,A.P. (1998) Genomic imprinting of a human apoptosis gene homologue, TSSC3. *Cancer Research*, 58, 1052-1056.

Li,J. et al. (2004) Imprinting of the human L3MBTL gene, a polycomb family member located in a region of chromosome 20 deleted in human myeloid malignancies. *Proceedings of the National Academy of Sciences of the United States of America*, 101, 7341-7346.

Meguro,M. et al. (1997) Evidence for uniparental, paternal expression of the human GABA(A) receptor subunit genes, using microcell-mediated chromosome transfer. *Human Molecular Genetics*, 6, 2127-2133.

Miyoshi,N. et al. (2000) Identification of an imprinted gene, Meg3/Gtl2 and its human homologue MEG3, first mapped on mouse distal chromosome 12 and human chromosome 14q. *Genes to Cells*, 5, 211-220.

Morison,I.M., Ramsay,J.P. and Spencer,H.G. (2005) A census of mammalian imprinting. *Trends in Genetics*, 21, 457-465.

Murphy,S.K., Wylie,A.A. and Jirtle,R.L. (2001) Imprinting of PEG3, the human homologue of a mouse gene involved in nurturing behavior. *Genomics*, 71, 110-117.

Nakabayashi,K. et al. (2004) Genomic imprinting of PPP1R9A encoding neurabin I in skeletal muscle and extra-embryonic tissues. *Journal of Medical Genetics*, 41, 601-608.

Ono,R. et al. (2003) Identification of a large novel imprinted gene cluster on mouse proximal chromosome 6. *Genome Research*, 13, 1696-1705.

Rachmilewitz,J. et al. (1992) Parental Imprinting of the Human H19 Gene. *Febs Letters*, 309, 25-28.

Shi,H.D. et al. (2003) Triple analysis of the cancer epigenome: An integrated microarray system for assessing gene expression, DNA methylation, and histone acetylation. *Cancer Research*, 63, 2164-2171.

Taniguchi,T., Okamoto,K. and Reeve,A.E. (1997) Human p57(KIP2) defines a new imprinted domain on chromosome 11p but is not a tumour suppressor gene in Wilms tumour. *Oncogene*, 14, 1201-1206.

Zhang,Y.H. and Tycko,B. (1992) Monoallelic Expression of the Human H19 Gene. *Nature Genetics*, 1, 40-44.

Zhang,Z. et al. (2006) Aberrant promoter methylation and silencing of the POU2F3 gene in cervical cancer. *Oncogene*, 25, 5436-5445.
