# Supplementary material for: Discovery of DNA methylation markers in cervical cancer using relaxation ranking
Source: BMC Med Genomics. 2008 Nov 24;1:57. doi: 10.1186/1755-8794-1-57 (PMC2605750; doi:10.1186/1755-8794-1-57)
Supplement: Additional file 6 — Supplementary table 4. enriched gene ontology terms, descriptions, number of genes associated with this GO term and P-value versus all human genes in the first 3000 probes. GO terms and statistics as determined by GOStat. [file 1755-8794-1-57-S6.doc]

Supplementary table 1: enriched gene ontology terms, descriptions, number of genes associated with this GO term and P-value versus all human genes in the first 3000 probes. GO terms and statistics as determined by GOStat

| 61BGene Ontology ID | 62BDescription | 63BNumber of genes | 64BP-value |
| --- | --- | --- | --- |
| GO:50789 | regulation of biological process | 470 | 4.69e-19 |
| GO:50791 | regulation of physiological process | 421 | 2.81e-18 |
| GO:50794 | regulation of cellular process | 443 | 2.81e-18 |
| GO:50875 | cellular physiological process | 1168 | 5.85e-18 |
| GO:51244 | regulation of cellular physiological process | 409 | 1.23e-17 |
| GO:9653 | morphogenesis | 91 | 1.21e-16 |
| GO:9987 | cellular process | 1354 | 3.71e-16 |
| GO:48513 | organ development | 75 | 5.44e-11 |
| GO:44238 | primary metabolism | 821 | 1.97e-09 |
| GO:8219 | cell death | 84 | 1.97e-09 |
| GO:16265 | death | 84 | 1.97e-09 |
| GO:6366 | transcription from RNA polymerase II promoter | 65 | 1.97e-09 |
| GO:6139 | nucleobase, nucleoside, nucleotide and nucleic acid metabolism | 419 | 2.04e-09 |
| GO:30154 | cell differentiation | 75 | 2.33e-09 |
| GO:7049 | cell cycle | 96 | 3.18e-09 |
| GO:19222 | regulation of metabolism | 305 | 5.64e-09 |
| GO:6355 | regulation of transcription, DNA-dependent | 265 | 6.32e-09 |
| GO:6351 | transcription, DNA-dependent | 269 | 1.03e-08 |
| GO:7399 | nervous system development | 61 | 1.48e-08 |
| GO:51242 | positive regulation of cellular physiological process | 66 | 1.63e-08 |
| GO:48731 | system development | 61 | 2.04e-08 |
| GO:44237 | cellular metabolism | 837 | 2.04e-08 |
| GO:48522 | positive regulation of cellular process | 74 | 2.44e-08 |
| GO:44255 | cellular lipid metabolism | 73 | 2.73e-08 |
| GO:31323 | regulation of cellular metabolism | 294 | 3.5e-08 |
| GO:6915 | apoptosis | 78 | 4.06e-08 |
| GO:43119 | positive regulation of physiological process | 67 | 4.06e-08 |
| GO:12501 | programmed cell death | 78 | 4.45e-08 |
| GO:6629 | lipid metabolism | 89 | 4.45e-08 |
| GO:6350 | transcription | 287 | 9.49e-08 |
| GO:7417 | central nervous system development | 23 | 1.02e-07 |
| GO:48518 | positive regulation of biological process | 81 | 1.17e-07 |
| GO:45449 | regulation of transcription | 277 | 1.4e-07 |
| GO:8152 | metabolism | 897 | 1.5e-07 |
| GO:19219 | regulation of nucleobase, nucleoside, nucleotide and nucleic acid metabolism | 278 | 3.1e-07 |
| GO:902 | cell morphogenesis | 41 | 1.02e-06 |
| GO:51726 | regulation of cell cycle | 61 | 1.44e-06 |
| GO:16043 | cell organization and biogenesis | 186 | 2.08e-06 |
| GO:74 | regulation of progression through cell cycle | 60 | 2.52e-06 |
| GO:42981 | regulation of apoptosis | 53 | 2.62e-06 |
| GO:43067 | regulation of programmed cell death | 53 | 3.43e-06 |
| GO:1501 | skeletal development | 23 | 5.37e-06 |
| GO:6357 | regulation of transcription from RNA polymerase II promoter | 37 | 9.74e-06 |
