# Supplementary material for: Discovery of DNA methylation markers in cervical cancer using relaxation ranking
Source: BMC Med Genomics. 2008 Nov 24;1:57. doi: 10.1186/1755-8794-1-57 (PMC2605750; doi:10.1186/1755-8794-1-57)
Supplement: Additional file 7 — Supplementary table 5. Overview of Ingenuity networks, highly represented in the top-3000 list. [file 1755-8794-1-57-S7.doc]

Supplementary table 1: overview of Ingenuity networks, highly represented in the top-3000 list

| 65BGenes | 66BScore (number of genes in top-3000, associated with pathway) | 67BFocus genes (total number of genes in pathway) | 68BPathway |
| --- | --- | --- | --- |
| AMID, ASPM, CABLES1, CABLES2, CCNA1, CHD3, CSTF1 (includes EG:1477), DGKA, DUSP2, GART, GBP1, GLIPR1, GSTM5, GTSE1, HSPA9B (includes EG:3313), KARCA1, KIAA1794, LPIN1, MAGEA4 (includes EG:4103), MCAM, PDRG1, PEG3 (includes EG:5178), PMS2, PODXL, PPP4R2, PTP4A1, RBBP6, RECQL4, RPRM, SNRK, TBX3, THRAP2, TP53, TRIO, VPS33A | 33 | 35 | Cancer, Cellular Compromise, DNA Replication, Recombination, and Repair |
| ACP1, AKAP12, CAD, CHRM2, CPE, CPEB1, DLGAP1, DUSP4, DUSP16, ELF1, FHL1, GNA11, GNAO1, GNB5, GRIK1, GRK6, INA, L1CAM (includes EG:3897), MAPK1, NEFH, NPDC1, NTS (includes EG:4922), PDE4D, PDE4DIP (includes EG:9659), PTPN7, PTPRR, RASD1, RGS7, SCLT1, SHANK2, SST, SSTR1, SYNGAP1, ULBP1, ULK1 | 33 | 35 | Cell Signaling, Nucleic Acid Metabolism, Small Molecule Biochemistry |
| ACOX1, ACTN2, API5, CIDEC, CNTFR, COL4A6, CTNND2, CYP24A1, DYRK1A, ERBB2IP, FGF2, FGFR4, GPC1, GREM1, GRIN2C, IL6ST, KCNAB1, LDB3, LIFR, LRRC7, MGAT5, MYPN, NR2C2, OLIG2, PDZD2, PI3, PTCRA, PTRF, SCD, SQSTM1, SYNPO, TEAD4, TGFB2, TGFBR3, ZNF148 | 33 | 35 | Cellular Development, Nervous System Development and Function, Reproductive System Development and Function |
| ANKS1B, BCL2, C1QL1, CASP9, CYCS, DCTN1, DCTN4, DNM1L, DYNLL1, ENDOG, GAST, GGA2, HK2, ITPR3, MAGEA3, NALP1, NALP2, NDE1, NNAT, NR4A1, NSF, PECAM1, PRKCE, PRKD2, PSEN2, PTGS2, RABEP1, RYR3, SLC25A4, TIMP3, TNK2, TRA@, TXNL5, UACA, WWOX | 33 | 35 | Cancer, Cell Death, Cell Morphology |
| ATF3, BCR, BMP2, BMP6, BMPER, COL1A1, CUL3, CYP7A1, EIF2AK4, FOSL1, FOSL2, FZD8, HGF, IGFBP5, JAG1, JUND, KLF9, KRR1, LDLR, MFI2, MLLT4, NFIX, NR2F1, PAPPA, PAPPA2, PCSK9, PTHR1, PTPN2, PVR, PVRL2, RHOD, RNF7, TMEM97, TRAM2, VTN | 33 | 35 | Organismal Development, Cellular Growth and Proliferation, Cellular Movement |
| AACS, ACACB, ACLY, ACSL1, ALDOC, CPT1A, DGAT1, DHCR7, FOXC2, GPX2, HMGCS1, HNF4A, IRS2, KRT20, LPL, LSS, MBTPS1, MUC13, NFATC4, PDK4, PDLIM7, PEG10, PPARG, PPARGC1A, PTGES, SCAND1, SCARB1, SLC16A1, SLC27A1, SLC27A2, SREBF1, SULT1A3, TIMM22, TPM2, UGT1A9 (includes EG:54600) | 33 | 35 | Lipid Metabolism, Molecular Transport, Small Molecule Biochemistry |
| ACD, AKAP13, ANK1, ARFGEF2, CAMK2N1, CGA, DNASE1, ECM1, H2AFX, HAND1, HMGA2, MAGED1, MEF2A, MUC5AC, NRGN, PDCD6, PRKAR1A, PRL, RECK, RHAG, S100A4, SFPQ, SH3GL2, SH3KBP1, SLC2A4RG, SND1 (includes EG:27044), SYNJ2, TDG, TERF2, THRA, TIAM1, TIMP2, TINF2, TNRC6A, XDH | 33 | 35 | Gene Expression, Organ Morphology, Reproductive System Disease |
| ARID4A, AYTL2, C1ORF181, CTH, DDX54, DNAJA2, EGR1, EGR4, FLI1, FLJ23584, GDF15, HDAC9, HIPK2, HSP90B1, IFRD1, IRS4, JARID1A, KLF1, LRCH4, MYEF2, NCOR1, PBX1, PEX6, PML, PRG1 (includes EG:5552), RARA, SF3A1, SF3A3, SIN3B, SMAD2, SMAD6, SMARCC2, TAT, VASN, ZBTB16 | 33 | 35 | Gene Expression, Cancer, Hematological Disease |
| CHP, DUSP8, DUSP19, EDAR, FGF12, GTF2I, ICAM1, IKBKG, MAP2K7, MAPK9, MDFIC, NFATC3, NFKB2, PDE8A, PRRX1, PSMB9, RAB6IP2, RALBP1, RIMS2, SCIN, SCN11A, SPN, STK4, STX7, TAOK2 (includes EG:9344), TNC, TNFAIP3, TNFRSF10B, TNFRSF10D, TP73L, TRAF1, TRAF2, TRAF3, TRAF4, TRIB3 | 33 | 35 | Cell Death, Connective Tissue Disorders, Cancer |
| ALG3, AURKA, CBX4, CCNB2, CDK10, CHFR, COX5A, COX7B2, COX8C, CTDSPL, DHFR, EIF4A1, ELN, FDFT1, GMDS (includes EG:2762), GPX3, HCN2, ID4, LZTR1, MGA (includes EG:23269), MXD4, MYBL2, NDUFC1, NEU1, NKX2-5, PCBP2, PDCD4, PLEC1, PTEN, SEC23A, SEC24A, SMOX, TFDP1, TNFAIP1, WDR45 (includes EG:11152) | 33 | 35 | Cell Cycle, Embryonic Development, Protein Synthesis |
| ANP32A, ATP2B1, ATXN1, BAT4, CUGBP1, CYP2C9, DLX1, DUSP1, ELK1, ELK4, EPB41L1, EYA2, FOXG1B, GAB1 (includes EG:2549), HELLS, INSR, ITGB8, ITPR1, ITSN1, KHDRBS2, KIFAP3, MXI1, PDE3B, PLCG1, PQBP1 (includes EG:10084), PRKCB1, PTPRG, SEZ6L2, SH2B1, SIX4, SIX5, SLC30A1, THPO, UNC5C, VIL1 | 33 | 35 | Gene Expression, Cancer, Cell Cycle |
| ATF1, CALR, CBS, CTGF, DAB2, DDAH1, DVL2, EPOR, FBLN1, GATA2, HLA-A, ITGB3, KIAA0020, LAMA4, LILRB2, MAGEA1, MAGEA2, MEF2C, MMP16, NAGA, NF1, NID2, NRG2, PCSK1, PCSK1N, PPAP2B, SDC4, SLA, TBCD, TCF3, TEAD1 (includes EG:7003), TGM2, TUBB4, TUBB2A, TWIST2 | 33 | 35 | Cardiovascular System Development and Function, Organismal Survival, Cell-To-Cell Signaling and Interaction |
| ARHGEF9, ARID1B (includes EG:57492), CALB1, CALD1, CD44, CDC42, CDC42BPA, CHM, CHML, DMD, FAH, IGF1R, KCNJ4, KCNJ12, KRAS, MKL2, MLLT10, MST1R, NF2, PIK3C2B, PIK3CA, PIP5K1A, RAB1A, RGS12, RPL35A, RPS24, RSN, SMARCA2, SNTG1, SPTA1, SPTBN1, SRF (includes EG:6722), SS18, VAV2, VAV3 | 33 | 35 | Cell Morphology, Cellular Assembly and Organization, Cellular Function and Maintenance |
| C2, C3, CACNA1A, CACNA1C, CR1, CSN2, DECR2, EPAS1, ERBB4, ETS2, HEXA, HSPA5, IGL@, IL12B, LLGL1, MASP1, MTA1, NEK1, NEK8, OSMR, PCLO, PKIB, PLD2, RAB40B, RNF41, SNAI1, SNAP25, SPINK4, STAT5B, STX3, STXBP1, SYT4, SYT9, TCEB2, WISP2 | 33 | 35 | Cellular Function and Maintenance, Organismal Injury and Abnormalities, Molecular Transport |
