# Supplementary material for: Discovery of DNA methylation markers in cervical cancer using relaxation ranking
Source: BMC Med Genomics. 2008 Nov 24;1:57. doi: 10.1186/1755-8794-1-57 (PMC2605750; doi:10.1186/1755-8794-1-57)
Supplement: Additional file 8 — Supplementary table 6. The ranking of possibly functional methylated genes from the highest-ranking probe-list (TOP250). Probes were ranked according the relaxation ranking algorithm ("original ranking"). Possible functionally methylated genes were selected ("new ranking") by omitting probes that do not fulfill the additional criteria. [file 1755-8794-1-57-S8.doc]

Supplementary table 1: The ranking of possibly functional methylated genes from the highest-ranking probe-list (TOP250). Probes were ranked according the relaxation ranking algorithm (“original ranking”). Possible functionally methylated genes were selected (“new ranking”) by omitting probes that do not fulfill the following criteria: (1) probes without gene symbol (i.e. gene ID) or hypothetical genes (marked as “unknown”); (2) probes/genes without a CpG island (marked as “no CpG”) because the expression of such markers is most probably reactivated upon DAC/TSA treatment indirectly via methylation-regulated transcription factors (Shi et al., 2003); (3) genes located on chromosome X (marked as “X-located”) since one of the main mechanisms of the inactivation of one copy of the X-chromosome in females is DNA methylation (see text); and (4) genes with expression that is not downregulated in less than 15 of the 39 carcinomas (marked as “untreated”); optimally no expression in all 39 cases is expected (P-call = 0), but relaxation ranking allows genes with varying P-calls including those that are expressed in more than 40% of all carcinomas). X, Y and Z represent the P-calls for primary tumor, untreated and treated cell lines, respectively.

| 69BOriginal rank | 70BNew rank | 71BAffy ID | 72BGene symbol | 73BChromosomal location | 74BX | 75BY | 76BZ | 77BReason for omitting |
| --- | --- | --- | --- | --- | --- | --- | --- | --- |
| 1 |  | 216370_s_at | TKTL1 | Xq28 | 0 | 0 | 12 | X-located |
| 2 |  | 211619_s_at | ALPP | 2q37 | 0 | 3 | 15 | No CpG |
| 3 | 1 | 206588_at | DAZL | 3p24.3 | 1 | 1 | 13 |  |
| 4 | 2 | 203865_s_at | ADARB1 | 21q22.3 | 1 | 2 | 15 |  |
| 5 |  | 210394_x_at | SSX4 | Xp11.23 | 2 | 1 | 14 | X-located |
| 6 |  | 235773_at | ZIK1 | 19q13.43 | 16 | 1 | 15 | Cancer |
| 7 |  | 214183_s_at | TKTL1 | Xq28 | 0 | 0 | 11 | Already selected |
| 8 | 3 | 1553599_a_at | SYCP3 | 12q | 0 | 1 | 12 |  |
| 9 |  | 220217_x_at | SPANXC | Xq27.1 | 1 | 2 | 14 | X-located |
| 10 |  | 243742_at | LOC388481 | 18q22.3 | 4 | 1 | 14 | Unknown |
| 11 |  | 220931_at | MGC5590 | 13q14.11 | 9 | 2 | 15 | Unknown |
| 12 |  | 1565909_at | - | - | 11 | 0 | 12 | Unknown |
| 13 |  | 211670_x_at | SSX3 | Xp11.23 | 0 | 0 | 10 | X-located |
| 14 |  | 204534_at | VTN | 17q11 | 0 | 2 | 12 | No CpG |
| 15 |  | 238825_at | ACRC | Xq13.1 | 2 | 1 | 13 | X-located |
| 16 |  | 204584_at | L1CAM | Xq28 | 5 | 3 | 15 | X-located |
| 17 |  | 222712_s_at | MUC13 | - | 9 | 1 | 14 | No CpG |
| 18 |  | 1563118_at | - | - | 10 | 0 | 11 | Unknown |
| 19 | 4 | 242721_at | AUTS2 | 7q11.22 | 12 | 0 | 12 |  |
| 20 |  | 1566656_a_at | RP11-151A6.2 | 13q32.3 | 0 | 0 | 9 | Unknown |
| 21 | 5 | 204239_s_at | NNAT | 20q11.2-q12 | 0 | 1 | 11 |  |
| 22 | 6 | 213921_at | SST | 3q28 | 1 | 1 | 12 |  |
| 23 | 7 | 226944_at | HTRA3 | 4p16.1 | 6 | 0 | 10 |  |
| 24 | 8 | 243161_x_at | ZFP42 | 4q35.2 | 11 | 1 | 14 |  |
| 25 |  | 236817_at | - | - | 15 | 0 | 12 | Unknown |
| 26 |  | 243802_at | DNHD2 | 3p14.3 | 23 | 2 | 15 | Cancer |
| 27 |  | 207493_x_at | SSX2 | Xp11.23-p11.22 | 0 | 0 | 8 | X-located |
| 28 |  | 220178_at | C19orf28 | 19p13.3 | 0 | 3 | 12 | Unknown |
| 29 | 9 | 204684_at | NPTX1 | 17q25.1-q25.2 | 2 | 3 | 14 |  |
| 30 |  | 231367_s_at | LOC647131 | 13q14.2 | 5 | 2 | 14 | Unknown |
| 31 |  | 215881_x_at | SSX2 | Xp11.23 | 11 | 0 | 11 | X-located |
| 32 | 10 | 1569555_at | GDA | 9q21.13 | 14 | 3 | 15 |  |
| 33 |  | 1561255_at | FAM50B | 6p25-pter | 27 | 0 | 12 | Cancer |
| 34 |  | 214035_x_at | LOC399491 | 16p13.1 | 31 | 2 | 15 | Cancer |
| 35 | 11 | 218720_x_at | SEZ6L2 | 16p11.2 | 1 | 3 | 13 |  |
| 36 |  | 229715_at | - | - | 1 | 3 | 13 | Unknown |
| 37 | 12 | 207013_s_at | MMP16 | 8q21 | 2 | 0 | 8 |  |
| 38 |  | 239343_at | - | - | 15 | 3 | 15 | Unknown |
| 39 |  | 202015_x_at | METAP2 | 12q22 | 35 | 2 | 15 | Cancer |
| 40 |  | 1556395_at | - | - | 0 | 1 | 10 | Unknown |
| 41 | 13 | 213711_at | KRTHB1 | 12q13 | 0 | 1 | 10 |  |
| 42 |  | 233938_at | FLJ22675 | 11q13.2 | 0 | 2 | 11 | No CpG |
| 43 |  | 243594_x_at | SPIRE2 | 16q24 | 0 | 2 | 11 | No CpG |
| 44 | 14 | 1553562_at | CD8B | 2p12 | 3 | 0 | 9 |  |
| 45 | 15 | 1570360_s_at | DDX3Y | Yq11 | 3 | 0 | 9 |  |
| 46 |  | 234516_at | - | - | 3 | 0 | 9 | Unknown |
| 47 |  | 1565641_at | C16orf45 | 16p13.11 | 3 | 1 | 12 | Unknown |
| 48 |  | 228404_at | IRX2 | 5p15.33 | 4 | 3 | 14 | Unknown |
| 49 |  | 224646_x_at | H19 | 11p15.5 | 31 | 1 | 14 | Cancer |
| 50 |  | 227721_at | CPAMD8 | 19p13.11 | 31 | 1 | 14 | Cancer |
| 51 |  | 1569987_at | DLEU7 | 13q14.3 | 1 | 1 | 11 | Unknown |
| 52 |  | 206626_x_at | SSX1 | Xp11.23-p11.22 | 1 | 1 | 11 | X-located |
| 53 |  | 1566927_at | C21orf104 | - | 5 | 2 | 13 | Unknown |
| 54 |  | 215595_x_at | GCNT2 | 6p24 | 11 | 0 | 10 | Unknown |
| 55 |  | 1554274_a_at | SSH1 | 12q24.11 | 17 | 3 | 15 | Cancer |
| 56 |  | 203549_s_at | LPL | 8p22 | 26 | 0 | 11 | Cancer |
| 57 |  | 215226_at | EXPH5 | 11q22.3 | 29 | 1 | 13 | Cancer |
| 58 |  | 229883_at | GRIN2D | 19q13.1-qter | 35 | 1 | 14 | Cancer |
| 59 |  | 211328_x_at | HFE | 6p21.3 | 37 | 2 | 15 | Cancer |
| 60 |  | 236486_at | - | - | 37 | 2 | 15 | Unknown |
| 61 | 16 | 234347_s_at | DENR | 12q24.31 | 8 | 2 | 13 |  |
| 62 | 17 | 227711_at | FAM112B | 12q13.2 | 14 | 2 | 14 |  |
| 63 |  | 1566110_at | - | - | 0 | 1 | 9 | Unknown |
| 64 |  | 211425_x_at | SSX4 | Xp11.23 | 0 | 1 | 9 | X-located |
| 65 |  | 232069_at | KIF26A | 14q32.33 | 0 | 3 | 11 | No CpG |
| 66 | 18 | 210021_s_at | UNG2 | 5p15.2-p13.1 | 2 | 3 | 13 |  |
| 67 | 19 | 1552785_at | FLJ37549 | 19q13.12 | 5 | 0 | 9 |  |
| 68 | 20 | 1553449_at | FLJ36701 | 16q24.3 | 5 | 0 | 9 |  |
| 69 |  | 1559441_s_at | LOC651430 | - | 5 | 0 | 9 | Unknown |
| 70 |  | 1559303_at | - | - | 5 | 1 | 12 | Unknown |
| 71 |  | 207739_s_at | GAGE1 | Xp11.4-p11.2 | 5 | 1 | 12 | X-located |
| 72 |  | 220051_at | PRSS21 | 16p13.3 | 18 | 3 | 15 | Cancer |
| 73 |  | 244503_at | BDNF | 11p13 | 18 | 3 | 15 | Cancer |
| 74 |  | 1566695_at | - | - | 30 | 0 | 11 | Cancer |
| 75 |  | 1557518_a_at | - | - | 0 | 0 | 7 | Unknown |
| 76 |  | 205749_at | CYP1A1 | 15q22-q24 | 0 | 0 | 7 | No CpG |
| 77 |  | 206549_at | INSL4 | 9p24 | 0 | 0 | 7 | No CpG |
| 78 |  | 206627_s_at | SSX1 | Xp11.23-p11.22 | 0 | 0 | 7 | No CpG |
| 79 |  | 208020_s_at | CACNA1C | 12p13.3 | 0 | 0 | 7 | No CpG |
| 80 |  | 210431_at | ALPPL2 | 2q37 | 0 | 0 | 7 | No CpG |
| 81 |  | 211731_x_at | SSX3 | Xp11.23 | 2 | 1 | 11 | No CpG |
| 82 | 21 | 201095_at | DAP | 5p15.2 | 6 | 3 | 14 |  |
| 83 | 22 | 206085_s_at | CTH | 1p31.1 | 6 | 3 | 14 |  |
| 84 |  | 239801_at | - | - | 7 | 1 | 12 | Unknown |
| 85 |  | 235009_at | FAM44A | 4p16.1 | 16 | 2 | 14 | Cancer |
| 86 |  | 207064_s_at | AOC2 | 17q21 | 20 | 3 | 15 | Cancer |
| 87 |  | 244451_x_at | TDG | 12q24.1 | 23 | 0 | 10 | Cancer |
| 88 |  | 234697_x_at | C3orf31 | 3p25.2 | 34 | 1 | 13 | Cancer |
| 89 |  | 204664_at | ALPP | 2q37 | 0 | 2 | 10 | No CpG |
| 90 |  | 238594_x_at | - | - | 0 | 2 | 10 | Unknown |
| 91 | 23 | 203548_s_at | LPL | 8p22 | 1 | 0 | 7 |  |
| 92 |  | 228713_s_at | DHRS10 | 19q13.33 | 32 | 0 | 11 | Cancer |
| 93 |  | 234401_at | - | - | 32 | 0 | 11 | Unknown |
| 94 |  | 1560477_a_at | SAMD11 | 1p36.33 | 1 | 3 | 12 | No CpG |
| 95 | 24 | 207147_at | DLX2 | 2q32 | 1 | 3 | 12 |  |
| 96 |  | 214612_x_at | MAGEA6 | Xq28 | 4 | 2 | 12 | X-located |
| 97 | 25 | 215672_s_at | KIAA0828 | 7q32.1 | 4 | 2 | 12 |  |
| 98 | 26 | 1570528_at | XYLT2 | 17q21.3-17q22 | 6 | 0 | 9 |  |
| 99 | 27 | 242763_at | PKHD1L1 | 8q23.1-q23.2 | 6 | 0 | 9 |  |
| 100 |  | 239153_at | FLJ41747 | 12q13.13 | 12 | 2 | 13 | No CpG |
| 101 |  | 229532_at | ZNF502 | 3p21.31 | 19 | 2 | 14 | Cancer |
| 102 |  | 242971_at | - | - | 19 | 2 | 14 | Unknown |
| 103 |  | 222890_at | HSPC065 | 16q21 | 24 | 3 | 15 | Cancer |
| 104 |  | 1558356_at | UACA | 15q22-q24 | 26 | 0 | 10 | Cancer |
| 105 |  | 215887_at | ZNF277 | 7q31.1 | 36 | 1 | 13 | Cancer |
| 106 |  | 206640_x_at | GAGE2 | Xp11.23 | 1 | 1 | 10 | X-located |
| 107 |  | 208155_x_at | GAGE4 | Xp11.4-p11.2 | 1 | 1 | 10 | X-located |
| 108 |  | 208235_x_at | GAGE7 | Xp11.4-p11.2 | 1 | 1 | 10 | X-located |
| 109 |  | 1563250_at | - | - | 4 | 1 | 11 | Unknown |
| 110 |  | 220021_at | TMC7 | 16p12.3 | 22 | 2 | 14 | Cancer |
| 111 |  | 1555141_a_at | LOC150159 | 4q24 | 35 | 0 | 11 | Cancer |
| 112 |  | 239582_at | PML | 15q22 | 35 | 0 | 11 | Cancer |
| 113 |  | 205554_s_at | DNASE1L3 | 3p21.1-3p14.3 | 39 | 2 | 15 | Cancer |
| 114 |  | 214218_s_at | XIST | Xq13.2 | 39 | 2 | 15 | Cancer |
| 115 |  | 217767_at | C3 | 19p13.3-p13.2 | 39 | 2 | 15 | Cancer |
| 116 |  | 218541_s_at | C8orf4 | 8p11.2 | 39 | 2 | 15 | Cancer |
| 117 |  | 223551_at | PKIB | 6q22.31 | 39 | 2 | 15 | Cancer |
| 118 |  | 226211_at | MEG3 | 14q32 | 39 | 2 | 15 | Cancer |
| 119 |  | 204637_at | CGA | 6q12-q21 | 0 | 1 | 8 | No CpG |
| 120 |  | 211403_x_at | VCY | Xp22.32 | 0 | 1 | 8 | X-located |
| 121 |  | 223365_at | DHX37 | 12q24.31 | 0 | 1 | 8 | No CpG |
| 122 |  | 237983_at | - | - | 0 | 1 | 8 | Unknown |
| 123 | 28 | 1566951_at | SIRT5 | 6p23 | 7 | 0 | 9 |  |
| 124 |  | 207851_s_at | INSR | 19p13.3-p13.2 | 7 | 0 | 9 | No CpG |
| 125 | 29 | 207069_s_at | SMAD6 | 15q21-q22 | 11 | 3 | 14 |  |
| 126 |  | 204959_at | MNDA | 1q22 | 39 | 0 | 11 | Cancer |
| 127 |  | 228988_at | ZNF6 | Xq21.1-q21.2 | 39 | 1 | 13 | X-located |
| 128 |  | 242907_at | GBP2 | 1p22.2 | 39 | 1 | 13 | Cancer |
| 129 | 30 | 1555788_a_at | TRIB3 | 20p13-p12.2 | 0 | 3 | 10 |  |
| 130 | 31 | 206290_s_at | RGS7 | 1q43 | 8 | 0 | 9 |  |
| 131 |  | 1557768_at | - | - | 14 | 1 | 12 | Unknown |
| 132 |  | 244378_at | C12orf51 | 12q24.13 | 25 | 2 | 14 | Cancer |
| 133 |  | 1569577_x_at | - | - | 26 | 3 | 15 | Unknown |
| 134 |  | 233185_at | ITGBL1 | 13q33 | 26 | 3 | 15 | Cancer |
| 135 |  | 239911_at | - | - | 26 | 3 | 15 | Unknown |
| 136 | 32 | 204717_s_at | SLC29A2 | 11q13 | 0 | 2 | 9 |  |
| 137 | 33 | 219480_at | SNAI1 | 20q13.1-q13.2 | 0 | 2 | 9 |  |
| 138 |  | 1562644_at | MTHFD2L | 4q13.3 | 2 | 0 | 7 | No CpG |
| 139 |  | 1565905_at | FLJ46026 | 17q25.3 | 2 | 0 | 7 | No CpG |
| 140 |  | 206371_at | FOLR3 | 11q13 | 2 | 0 | 7 | No CpG |
| 141 |  | 220348_at | KBTBD9 | 2p24.1 | 2 | 0 | 7 | No CpG |
| 142 | 34 | 1564066_at | SPATS1 | 6p21.1 | 6 | 1 | 11 |  |
| 143 | 35 | 207151_at | ADCYAP1R1 | 7p14 | 9 | 0 | 9 |  |
| 144 |  | 229685_at | TBC1D14 | 4p16.1 | 13 | 3 | 14 | No CpG |
| 145 |  | 241252_at | ESCO2 | 8p21.1 | 13 | 3 | 14 | No CpG |
| 146 |  | 231023_at | FLJ12118 | 13q34 | 14 | 2 | 13 | Unknown |
| 147 | 36 | 235872_at | TERF2 | 16q22.1 | 14 | 2 | 13 |  |
| 148 |  | 239984_at | SCN7A | 2q21-q23 | 14 | 2 | 13 | No CpG |
| 149 |  | 232324_x_at | PPAP2B | 1pter-p22.1 | 28 | 3 | 15 | Cancer |
| 150 | 37 | 242138_at | DLX1 | 2q32 | 2 | 3 | 12 |  |
| 151 |  | 1557873_at | SPINK5L3 | 5q33.1 | 5 | 2 | 12 | No CpG |
| 152 |  | 229932_at | LOC51255 | 2p11.2 | 7 | 0 | 8 | Unknown |
| 153 |  | 1552858_at | MAGEB6 | Xp21.3 | 15 | 1 | 12 | X-located |
| 154 |  | 214230_at | CDC42 | 1p36.1 | 15 | 2 | 13 | Cancer |
| 155 |  | 1557193_at | PTPN2 | 18p11.3-p11.2 | 28 | 2 | 14 | Cancer |
| 156 |  | 208591_s_at | PDE3B | 11p15.1 | 28 | 2 | 14 | Cancer |
| 157 |  | 242153_at | LARP2 | 4q28.2 | 33 | 0 | 10 | Cancer |
| 158 |  | AFFX-DapX-3_at | - | - | 0 | 4 | 15 | Unknown |
| 159 |  | AFFX-DapX-0_at | - | - | 0 | 4 | 15 | Unknown |
| 160 |  | AFFX-LysX-3_at | - | - | 0 | 4 | 15 | Unknown |
| 161 |  | AFFX-LysX-5_at | - | - | 0 | 4 | 15 | Unknown |
| 162 |  | AFFX-1heX-3_at | - | - | 0 | 4 | 15 | Unknown |
| 163 |  | AFFX-1heX-5_at | - | - | 0 | 4 | 15 | Unknown |
| 164 |  | AFFX-1heX-0_at | - | - | 0 | 4 | 15 | Unknown |
| 165 |  | AFFX-ThrX-3_at | - | - | 0 | 4 | 15 | Unknown |
| 166 |  | AFFX-ThrX-5_at | - | - | 0 | 4 | 15 | Unknown |
| 167 |  | AFFX-ThrX-0_at | - | - | 0 | 4 | 15 | Unknown |
| 168 |  | AFFX-r2-Bs-dap-3_at | - | - | 0 | 4 | 15 | Unknown |
| 169 |  | AFFX-r2-Bs-dap-5_at | - | - | 0 | 4 | 15 | Unknown |
| 170 |  | AFFX-r2-Bs-dap-0_at | - | - | 0 | 4 | 15 | Unknown |
| 171 |  | AFFX-r2-Bs-lys-5_at | - | - | 0 | 4 | 15 | Unknown |
| 172 |  | AFFX-r2-Bs-lys-0_at | - | - | 0 | 4 | 15 | Unknown |
| 173 |  | AFFX-r2-Bs-phe-5_at | - | - | 0 | 4 | 15 | Unknown |
| 174 |  | AFFX-r2-Bs-phe-0_at | - | - | 0 | 4 | 15 | Unknown |
| 175 |  | AFFX-r2-Bs-thr-0_s_at | - | - | 0 | 4 | 15 | Unknown |
| 176 |  | 1569208_a_at | DKFZP686A01247 | 4p13 | 2 | 2 | 11 | No CpG |
| 177 |  | 228066_at | LOC642705 | 17q12 | 2 | 2 | 11 | No CpG |
| 178 |  | 240983_s_at | CARS | 11p15.5 | 2 | 2 | 11 | No CpG |
| 179 |  | 210675_s_at | PTPRR | 12q15 | 6 | 3 | 13 | No CpG |
| 180 | 38 | 218476_at | POMT1 | 9q34.1 | 6 | 3 | 13 |  |
| 181 |  | 1562311_at | - | - | 7 | 1 | 11 | Unknown |
| 182 |  | 1557558_s_at | MATN1 | 1p35 | 29 | 2 | 14 | Cancer |
| 183 |  | 220187_at | STEAP4 | 7q21.12 | 29 | 3 | 15 | Cancer |
| 184 |  | 227045_at | ZNF614 | 19q13.33 | 29 | 3 | 15 | Cancer |
| 185 |  | AFFX-r2-Bs-thr-3_s_at | - | - | 0 | 4 | 13 | Unknown |
| 186 |  | 207086_x_at | GAGE2 | Xp11.23 | 2 | 1 | 10 | X-located |
| 187 |  | 215317_at | - | - | 2 | 1 | 10 | Unknown |
| 188 |  | 239369_at | LCN8 | 9q34.3 | 2 | 1 | 10 | No CpG |
| 189 | 39 | 203731_s_at | ZFP95 | 7q22 | 14 | 3 | 14 |  |
| 190 |  | 206776_x_at | ACRV1 | 11p12-q13 | 24 | 1 | 12 | Cancer |
| 191 |  | 208511_at | PTTG3 | 8q13.1 | 30 | 2 | 14 | Cancer |
| 192 |  | 1553508_at | MDS2 | 1p36 | 1 | 1 | 9 | No CpG |
| 193 |  | 204927_at | RASSF7 | 11p15.5 | 1 | 1 | 9 | Unknown |
| 194 |  | 207663_x_at | GAGE3 | Xp11.4-p11.2 | 1 | 1 | 9 | X-located |
| 195 | 40 | 229839_at | SCARA5 | 8p21.1 | 1 | 1 | 9 |  |
| 196 |  | AFFX-r2-Bs-phe-3_at | - | - | 2 | 4 | 15 | Unknown |
| 197 | 41 | 223954_x_at | APBA2BP | 20q11.22 | 3 | 2 | 11 |  |
| 198 | 42 | 1568981_at | GTF2I | 7q11.23 | 7 | 3 | 13 |  |
| 199 |  | 238729_x_at | LOC646561 | 4q31.22 | 7 | 3 | 13 | No CpG |
| 200 | 43 | 1569998_at | MMD2 | 7p22.1 | 9 | 1 | 11 |  |
| 201 |  | 1557890_at | LOC645818 | 6q24.3 | 16 | 2 | 13 | Cancer |
| 202 |  | 241368_at | - | - | 30 | 3 | 15 | Unknown |
| 203 |  | 241719_at | - | - | 30 | 3 | 15 | Unknown |
| 204 |  | 1554633_a_at | MYT1L | 2p25.3 | 1 | 3 | 11 | No CpG |
| 205 |  | 206084_at | PTPRR | 12q15 | 1 | 3 | 11 | Already selected |
| 206 |  | 1552829_at | TMEM23 | 10q11.2 | 25 | 1 | 12 | Cancer |
| 207 |  | 207059_at | PAX9 | 14q12-q13 | 25 | 1 | 12 | Cancer |
| 208 |  | 1569600_at | DLEU2 | 13q14.3 | 38 | 0 | 10 | Cancer |
| 209 |  | 244313_at | CR1 | 1q32 | 38 | 0 | 10 | Cancer |
| 210 |  | 210016_at | MYT1L | 2p25.3 | 0 | 3 | 9 | Already selected |
| 211 |  | 219184_x_at | - | 17p13 | 0 | 3 | 9 | Unknown |
| 212 |  | 239267_at | NEK6 | 9q33.3-q34.11 | 5 | 4 | 15 | Untreated |
| 213 |  | 1568639_a_at | - | - | 8 | 3 | 13 | Unknown |
| 214 |  | 239302_s_at | - | - | 9 | 2 | 12 | Unknown |
| 215 |  | 1556387_at | - | - | 17 | 2 | 13 | Unknown |
| 216 |  | 216450_x_at | HSP90B1 | 12q24.2-q24.3 | 17 | 2 | 13 | Cancer |
| 217 |  | 1568830_at | RBMS1 | 2q24.2 | 26 | 1 | 12 | Cancer |
| 218 |  | 1552906_at | FMR1NB | Xq27.3-q28 | 0 | 0 | 6 | X-located |
| 219 |  | 1564854_at | - | - | 0 | 0 | 6 | Unknown |
| 220 |  | 1565836_at | - | - | 0 | 0 | 6 | Unknown |
| 221 | 44 | 1567341_at | FOXD4 | 9p11-q11 | 0 | 0 | 6 |  |
| 222 |  | 220921_at | SPANXB1 | Xq27.1 | 0 | 0 | 6 | X-located |
| 223 |  | 229895_s_at | NCK1 | 3q21 | 0 | 0 | 6 | Unknown |
| 224 |  | 231307_at | PAGE2 | Xp11.21 | 0 | 0 | 6 | X-located |
| 225 |  | 235700_at | RP13-36C9.1 | Xq26.3 | 0 | 0 | 6 | X-located |
| 226 |  | 237580_at | - | - | 0 | 0 | 6 | Unknown |
| 227 |  | 238568_s_at | C18orf8 | 18q11.2 | 0 | 0 | 6 | Unknown |
| 228 | 45 | 238607_at | ZNF342 | 19q13.32 | 0 | 0 | 6 |  |
| 229 | 46 | 240313_at | DMRTB1 | 1p32.3 | 0 | 0 | 6 |  |
| 230 |  | 243171_at | - | - | 0 | 0 | 6 | Unknown |
| 231 |  | 1561705_at | TTBK2 | 15q15.2 | 0 | 2 | 8 | Unknown |
| 232 |  | 207214_at | SPINK4 | 9p13.3 | 0 | 2 | 8 | No CpG |
| 233 |  | 1556107_at | - | - | 3 | 0 | 7 | Unknown |
| 234 | 47 | 205899_at | CCNA1 | 13q12.3-q13 | 3 | 0 | 7 |  |
| 235 |  | 221112_at | IL1RAPL2 | Xq22.2-q22.3 | 3 | 0 | 7 | X-located |
| 236 | 48 | 233490_at | DCTN4 | 5q31-q32 | 3 | 0 | 7 |  |
| 237 |  | 1566823_a_at | - | - | 3 | 1 | 10 | Unknown |
| 238 |  | 226516_at | C19orf28 | 19p13.3 | 3 | 1 | 10 | Already selected |
| 239 | 49 | 229475_at | MAEL | 1q24.1 | 3 | 1 | 10 |  |
| 240 |  | 223575_at | KIAA1549 | 7q34 | 15 | 3 | 14 | Cancer |
| 241 |  | 233931_at | ELF1 | 13q13 | 15 | 3 | 14 | Cancer |
| 242 |  | 236646_at | C12orf59 | 12p13.2 | 15 | 3 | 14 | Cancer |
| 243 |  | 207660_at | DMD | Xp21.2 | 18 | 2 | 13 | X-located |
| 244 |  | 219922_s_at | LTBP3 | 11q12 | 31 | 2 | 14 | Cancer |
| 245 | 50 | 1552368_at | CTCFL | 20q13.31 | 14 | 0 | 9 |  |
| 246 |  | 1552414_at | WFDC9 | 20q12-q13.1 | 14 | 0 | 9 | No CpG |
| 247 |  | 1562674_at | - | - | 14 | 0 | 9 | Unknown |
| 248 |  | 205876_at | LIFR | 5p13-p12 | 32 | 3 | 15 | Cancer |
| 249 |  | 210233_at | IL1RAP | 3q28 | 32 | 3 | 15 | Cancer |
| 250 |  | 215606_s_at | RAB6IP2 | 12p13.3 | 32 | 3 | 15 | Cancer |

^1^: Cancer means “too many cancers” positive and therefore not selected based on the set criteria.
^2^: Untreated means that in the untreated cell Lines already all cell Lines are positive and therefore not selected based on the set criteria.
